# MOSS—Multi-Modal Best Subset Modeling in Smart Manufacturing

**DOI:** 10.3390/s21010243

**Published:** 2021-01-01

**Authors:** Lening Wang, Pang Du, Ran Jin

**Affiliations:** 1 Grado Department of Industrial and Systems Engineering, Virginia Tech, Blacksburg, VA 24061, USA; wangln@vt.edu; 2 Department of Statistics, Virginia Tech, Blacksburg, VA 24061, USA; pangdu@vt.edu

**Keywords:** data fusion, fused deposition modeling, multi-modal sensing, quality modeling, smart manufacturing

## Abstract

Smart manufacturing, which integrates a multi-sensing system with physical manufacturing processes, has been widely adopted in the industry to support online and real-time decision making to improve manufacturing quality. A multi-sensing system for each specific manufacturing process can efficiently collect the in situ process variables from different sensor modalities to reflect the process variations in real-time. However, in practice, we usually do not have enough budget to equip too many sensors in each manufacturing process due to the cost consideration. Moreover, it is also important to better interpret the relationship between the sensing modalities and the quality variables based on the model. Therefore, it is necessary to model the quality-process relationship by selecting the most relevant sensor modalities with the specific quality measurement from the multi-modal sensing system in smart manufacturing. In this research, we adopted the concept of best subset variable selection and proposed a new model called Multi-mOdal beSt Subset modeling (MOSS). The proposed MOSS can effectively select the important sensor modalities and improve the modeling accuracy in quality-process modeling via functional norms that characterize the overall effects of individual modalities. The significance of sensor modalities can be used to determine the sensor placement strategy in smart manufacturing. Moreover, the selected modalities can better interpret the quality-process model by identifying the most correlated root cause of quality variations. The merits of the proposed model are illustrated by both simulations and a real case study in an additive manufacturing (i.e., fused deposition modeling) process.

## 1. Introduction

Smart manufacturing integrates multi-modal sensing systems and computing resources (e.g., Fog computing and Cloud computing) to support efficient real-time quality modeling, monitoring, diagnosis, and control in manufacturing [1,2,3,4]. Specifically, one modality in this paper is defined as a group of features extracted from the sensing signal that measures the same kind of physical quantity from the same place in the manufacturing process [5]. Therefore, based on the multi-modal sensing systems, variables that can reflect the status of manufacturing processes are collected from different modalities to effectively model the quality-process relationship in smart manufacturing [6,7]. However, how to effectively design and achieve the multi-modal sensing system in smart manufacturing is still an open question [8]. For example, one can equip sensors and collect the corresponding process variables as many as possible to accurately model the quality-process relationship in the manufacturing process. But this approach is not cost-effective, because some modalities might be redundant or comparable with each other. On the other hand, with a multi-modal sensing system, it is important to identify the most relevant modalities in a quality-process model to effectively interpret the potential root cause of the quality variation [9]. Moreover, from the sensitivity analysis perspective, it is important to identify how the model will be changed with or without a specific sensor modality, and how the modality contributes to modeling the response. Therefore, it is critical to find a quality-process model strategy that can effectively select the best subset from the multi-modal sensing data, and rank the relevance for each modality toward the modeled quality variable.

**Figure 1:**
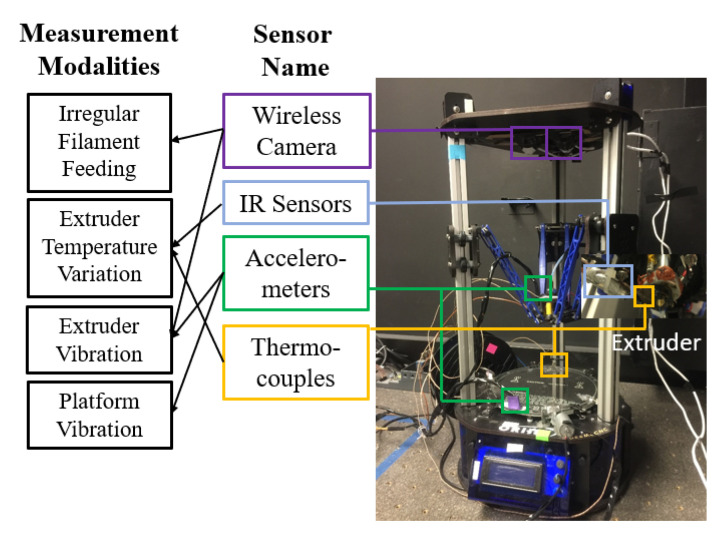
A Delta fused deposition modeling (FDM) Printer with a Multi-Modal Sensing System (Redrawn from [18] with author’s permission).

Take the fused deposition modeling (FDM), which is an extruder based additive manufacturing (AM) process, as an example [10]. As a promising advanced manufacturing process, FDM can efficiently fabricate personalized products with a high degree of geometric complexity [11,12,13]. and has been employed in many significant applications [14,15]. However, most of these applications are not yet widely deployed in practice due to the quality variation of products, such as geometric deviations [16,17]. Because the fabrication mechanism of the FDM process is complex, the potential root cause for the geometric deviation is also diverse. As shown in Figure 1 [18], in order to comprehensively study how the anomaly can influence the geometric deviations, a smart manufacturing paradigm of the FDM process with a multi-modal sensing system is proposed. The data collected from these sensor modalities can directly or indirectly reflect the characteristics and variations of the fabrication in the FDM process. However, this design for the multi-modal sensing system might not be the most cost-effective. For example, the data collected from the infrared sensor and the thermocouple on the extruder might be correlated since both of them measure the thermal distribution near the melting pool area [12]. In the literature, there has been a series of quality-process models to study the influence of different sensor modalities on the quality variable [10,12,19,20,21]. However, most of the existing quality-process models cannot work for nonlinear model components, and thus cannot identify the significant modalities to obtain a cost-effective (e.g., without redundant or comparable modalities) multi-modal sensing system. Moreover, the interpretability of these quality-process models might be questionable, without identifying the significant sensing modalities and ranking their contributions toward a specific quality variable in the FDM process. Therefore, it is important to quantify the relevance of each sensor modality toward the specific quality response in quality modeling. In this way, we can provide a cost-effective multi-modal sensing system to the FDM process, and also accurately pinpoint the potential root cause of a defect based on the sensor modality selection result to reduce or avoid the product defect in the future [10].

The objective of this research is to propose a model that can effectively select the real-time sensing modalities in quality modeling to support the cost-effective multi-modal sensing system design in smart manufacturing. To tackle the knowledge gap, we propose a new modeling method called Multi-mOdal beSt Subset modeling (MOSS) that adopts the best subset selection idea from the best subset regression [22]. The proposed MOSS can effectively select the best subset from the original data set via a two-level variable selection (i.e., among sensor modalities and within each modality) effort. Specifically, two regularization norms are embedded in the quality-process model to realize this effort. The first one is a functional norm that can effectively identify the relevance of each sensor modality toward the quality response in model estimation. Smoothing splines framework [23] is used to represent nonlinear model components, and quantify the contribution of each modality in the proposed MOSS. By comparing the magnitudes of functional norms among modalities estimated from the model, the rank of relevance toward the quality response can be accurately identified. The second norm is an *l*-1 norm that encourages the sparsity of model coefficients corresponding to features within each data modality. By comparing with the existing methods [24,25,26,27,28], the proposed MOSS can realize the two-level variable selection simultaneously with both linear and nonlinear model components, and further select the sensor modalities in smart manufacturing. To evaluate the quality prediction performance and the variable selection accuracy for the proposed MOSS, both simulations and a real case study are implemented. The results show the proposed MOSS can effectively select the significant modalities with an accurate variable selection accuracy via the smooth spline framework compared with three benchmark methods (i.e., Lasso regression [24], group Lasso [25], and hierarchical Lasso [26]).

The rest of the paper is organized as follows—Section 2 summarizes the state-of-the-art of quality improvement and modeling for FDM processes and multi-modal modeling methods. Section 3 introduces the proposed MOSS model in detail. Section 4 validates the prediction performance and the variable selection accuracy of the proposed method via a simulation study. Section 5 employs a real case study on the FDM process to model multiple geometric quality measurements via the proposed MOSS. Lastly, Section 6 concludes and discusses future work.

## 2. Related Works

In this section, the state-of-the-art research on quality improvement and modeling for the AM process is reviewed. First, to improve the product quality from the AM process, the optimized process recipe (i.e., the combination of process setting variables) has been studied. For example, Fordan et al. identified how the important setting variables (e.g., layer thickness) can influence the mechanical property of the AM products through a design of experiment study [29]. Moreover, for the geometric deviation of the product, Sood et al. employed the gray Taguchi method to study the influence of five setting variables (i.e., part orientation, deposition width, layer thickness, air gap, and deposition angle) on the product geometric deviation [30]. Similarly, Zhang and Peng applied the Taguchi method which is combined with a fuzzy comprehensive evaluation to established empirical relations between the setting variables and the geometric deviation of product [31]. However, the aforementioned works mainly concentrate on the run-to-run study to optimize the process recipe and identify the significant process setting variables in the AM process, instead of modeling the relationship between the product quality with the process variables from the sensing system which can reflect the real-time fabrication variation.

To model the in situ sensing data with the product in an AM process, many data-driven models have been proposed in the literature. For instance, Rao et al. presented an advanced Bayesian nonparametric analysis method for in situ sensing data to identified process failures and the types of failures in a FDM process in real-time [10]. Sun et al. proposed a functional quantitative and qualitative model to predict two types of quality responses via offline setting variables and in situ process variables [12]. Tlegenov et al. presented a nozzle clogging monitoring system based on the in situ vibration data through a physics-based dynamic model for a FDM process [32]. Li et al. proposed a data-driven method for in situ monitoring and process diagnosis based on the vibration sensors. The least-squares support vector machine (LS-SVM) method was employed to identify the filament clogging event in real-time. Liu et al. proposed a data-driven model to predict the product surface roughness based on the features generated from thermocouples, infrared temperature sensors, and accelerometers [33]. Kousiatza and Karalekas illustrated a geometric deviation monitoring system based on the fiber Bragg grating sensors and thermocouples. The in situ data collected from the sensors is employed to generate the temperature distribution and product profile based on a data-driven model [34]. However, the aforementioned methods typically only focus on quality-process modeling instead of selecting the relevance of sensing modality. Thus, they may not provide insights on the contribution of each sensing modality toward the quality variable. Therefore, the existed method might be not sufficient to guide the multi-modal sensing system design in smart manufacturing.

On the other hand, there are many modality and variable selection modeling methods that have been proposed in the literature [35]. For example, Tibshirani proposed the Lasso penalty to employ the variable selection effort in an ordinary regression model by constraining the sum of the absolute value of the model coefficients being less than a constant [24]. To extend the variable selection efforts for the different modality of predictors, the group Lasso was proposed [36]. The group Lasso proposed a group-wise penalty to encourage the group (i.e., data modality) sparsity in model estimation. To effectively implement the modality selection and the variable selection within each modality simultaneously, Huang et al. proposed the group bridge method to simultaneously select the important modality and also the feature within each modality at the same time via a specially designed group bridge penalty [37]. However, the proposed group bridge penalty is not always differentiable and tends to be inconsistent for feature selection [38]. Zhou and Zhu proposed the hierarchical Lasso approach to effectively remove insignificant modality and implement the variable selection within each modality by penalizing the coefficients using two levels of *l*-1 penalty [26]. Paynabar et al. [27] and Sun et al. [28] proposed a hierarchical nonnegative garrote method to achieve these two-level variable selection efforts in linear regression models. Fan and Li developed the smoothly clipped absolute deviation (SCAD) penalty to effectively select variables and estimate linear model coefficients simultaneously [39]. However, the aforementioned methods mainly focus on selecting linear functional model components, and cannot deal with the nonlinear model components. For the nonlinear model components, Lin and Zhang proposed the component Selection and Smoothing Operator (COSSO) method to regularize the data modality as the summation of component norms based on the smooth spline method [40]. Ravikumar et al. proposed the sparse additive model (SpAM) to regularize the data modality based on an empirical functional norm via a non-parametric smoother [41]. However, these methods do not involve the variable selection effort within each modality among the nonlinear model components. Therefore, it is important to propose a model that can handle the nonlinear model components with the capability that can simultaneously select both the significant modalities and the variables within each modality in model estimation.

## 3. Methodology

**Figure A1:**
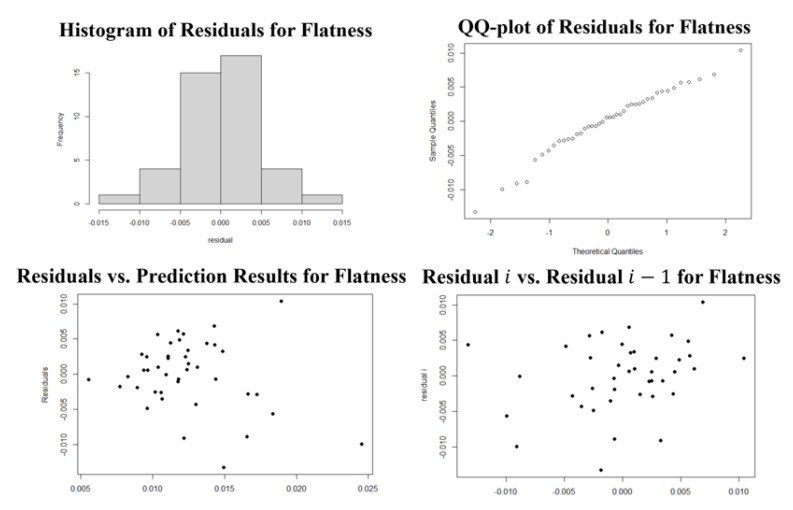
Residual Plot and Assumption Check for the Proposed MOSS in Case Study.

In order to clarify the scope of this study, we assume that an additive model structure is sufficient to model the quality-process relationship. This assumption is validated in Figure A1 in Appendix A. Moreover, in order to reduce the spatio-temporal data registration complexity and the corresponding intensive computation loads of model estimation, the overall geometric quality variable for product *i* is treated as the output in modeling, denoted as $y_{i}$ and i=1,⋯,n. The model can be expressed as:

(1) $$y_{i}=\alpha +\sum_{r=1}^{d}f_{r}\left(x_{ir}^{T}\beta_{r}\right)+\epsilon_{i},$$

where α is an unknown intercept, $f_{r}$’s are unknown smooth functions, $x_{ir}={\left(x_{ir1},\ldots ,x_{irp_{r}}\right)}^{T}$ is the feature vector generated from modality *r* for product *i* with $p_{r}$ number of features, and $\beta_{r}={\left(\beta_{r1},\ldots ,\beta_{rp_{r}}\right)}^{T}$ is the vector of weight coefficients for the predictor vector $x_{ir}$. It is worth to mention that the data can be aligned based on the dynamic time warping [42]. To guarantee that model Equation (1) is estimable, in this paper, we shall use the constraints $\int f_{r}=0,r=1,\ldots ,d$ [43]. Therefore the quality-process relationship in Equation (1) can be expressed as an additive model where each modality is represented by an additive component function $f_{r}$. This model structure can help better interpret the contribution of each modality component [9]. The proposed model framework can potentially be extended to multidimensional output with a proper spatio-temporal registration strategy and an appropriate model structure to estimate the spatio-temporal effects, such as functional regression [44]. To estimate component function $f_{r}$, $f_{r}$ is formulated in a reproducing kernel Hilbert space (RKHS) framework. Specifically, the whole mean response function ($\alpha +\sum_{r=1}^{d}f_{r}$) in Equation (1) is assumed to reside in an RKHS F of functions. The space has a tensor sum decomposition $F=1⨁F_{1}$ with $F_{1}={⨁}_{r=1}^{d}F^{r}$, where $F^{1},\cdots ,F^{d}$ are *d* orthogonal subspaces of F such that $f_{r}\in F^{r}$ to indicate *d* modalities. To estimate the model parameters $\left(f_{r},\alpha ,\beta_{r}\right)$, a penalized least square optimization formulation is proposed as:

(2) $$\underset{f_{r},\beta_{r}}{\mathrm{argmin}}\sum_{i}{\left(y_{i}-\alpha -\sum_{r=1}^{d}f_{r}\left(x_{ir}^{T}\beta_{r}\right)\right)}^{2}+\lambda_{1}\sum_{r}{∥\beta_{r}∥}_{1}+\lambda_{2}\sum_{r=1}^{d}{∥f_{r}∥}_{2},$$

where the first term $\sum_{i}{\left(y_{i}-\alpha -\sum_{r=1}^{d}f_{r}\left(x_{ir}^{T}\beta_{r}\right)\right)}^{2}$ represents the least-square loss for model estimation; $\sum_{r}{∥\beta_{r}∥}_{1}=\sum_{r}\sum_{j=1}^{p_{r}}\left|\beta_{rj}\right|$ is the *l*-1 regularization term which implements the variable selection effort within each modality [24]; $\lambda_{1}$ is the tuning parameter to control the sparsity of the $\beta_{r}$; $\sum_{r=1}^{d}{∥f_{r}∥}_{2}=\sum_{r=1}^{d}\sqrt{\int f_{r}^{2}}$ is the *L*-2 functional norm regularization to determine the sparsity among data modalities [37]. Therefore, the proposed MOSS can effectively and simultaneously select the significant sensing modalities for nonlinear function components, and also identify the important predictors within each modality. To effectively estimate the functional norm for each modality, modality inputs $x_{ir}^{T}\beta_{r}$, i=1,⋯,n, are all standardized to [0,1] within each modality. Therefore, by comparing the magnitude of functional norms, the best subset of modalities toward the quality response can be effectively identified. It is worth to mention that, once the significant modalities and the important features within each modality are identified, the raw sensor features can be used to interpret the root cause of the product defects. Moreover, by choosing the different tuning parameter $\lambda_{2}$, the number of selected modalities in the best subset can be controlled.

To estimate the model parameters in Equation (2), a block updating algorithm is developed to break down the proposed optimization problem into two simpler optimization problems as follow:

(3) $$\underset{\alpha ,f_{r}}{\mathrm{argmin}}\sum_{i}{\left(y_{i}-\alpha -\sum_{r=1}^{d}f_{r}\left(x_{ir}^{T}\beta_{r}\right)\right)}^{2}+\lambda_{2}\sum_{r=1}^{d}{∥f_{r}∥}_{2},$$

and

(4) $$\underset{\beta_{r}}{\mathrm{argmin}}\sum_{i}{\left(y_{i}-\alpha -\sum_{r=1}^{d}f_{r}\left(x_{ir}^{T}\beta_{r}\right)\right)}^{2}+\lambda_{1}\sum_{r}{∥\beta_{r}∥}_{1},$$

a direct optimization of Equation (3) is difficult due to the functional norm regularization term. Inspired by the COmponent Selection and Smoothing Operator (COSSO) [40], an equivalent formulation of Equation (3) is proposed as follow:

(5) $$\underset{\alpha ,f_{r},\theta_{r}}{\mathrm{argmin}}\sum_{i}{\left(y_{i}-\alpha -\sum_{r=1}^{d}f_{r}\left(x_{ir}^{T}\beta_{r}\right)\right)}^{2}+\lambda_{0}\sum_{r=1}^{d}\theta_{r}^{-1}{∥f_{r}∥}_{2}^{2}+\lambda_{2}\sum_{r=1}^{d}\theta_{r},$$

where $\lambda_{0}$ is a tuning constant and $\theta_{r}⩾0$ is the constrained weight coefficients for each sensor modality. By the representer theorem for smooth splines [23], the solution of $f_{r}$ has the form $f_{r}\left(x\right)=\sum_{i=1}^{n}c_{i}\theta_{r}R_{r}\left(x_{ir}^{T}\beta_{r},x\right)$, where $c_{i}$’s are unknown coefficients and $R_{r}$ is the reproducing kernel function of $F^{r}$. Let $R_{r}^{*}$ be the n×n matrix with the (i,j)-th element being $R_{r}\left(\left(x_{ir}^{T}\beta_{r}\right),\left(x_{jr}^{T}\beta_{r}\right)\right)$, i=1,⋯,n, j=1,⋯,n. Define $R_{\theta }=\sum_{r=1}^{d}\theta_{r}R_{r}$ and the matrix $R_{\theta }^{*}=\sum_{r=1}^{d}\theta_{r}R_{r}^{*}$. For fixed $\theta_{r}$’s, we can find the estimates of the intercept α and the coefficient vector $c={\left(c_{1},\cdots ,c_{n}\right)}^{T}$ by

(6) $$\underset{\alpha ,c}{\mathrm{argmin}}{\left(y-\alpha 1_{n}-R_{\theta }^{*}c\right)}^{T}\left(y-\alpha 1_{n}-R_{\theta }^{*}c\right)+n\lambda_{0}c^{T}R_{\theta }^{*}c,$$

which is a standard smoothing spline problem [23] and can be solved, including the tuning of $\lambda_{0}$, by standard smoothing splines software [43]. By fixing α and c, defining $g_{r}=R_{r}^{*}c$ and letting *G* be the n×r matrix with the *r*-th column being $g_{r}$, we can efficiently solve $\theta ={\left(\theta_{1},\cdots ,\theta_{d}\right)}^{T}$ by

(7) $$\underset{\theta }{\mathrm{argmin}}{\left(z-G\theta \right)}^{T}\left(z-G\theta \right)+n\lambda_{2}\sum_{r=1}^{d}\theta_{r},\;\;\mathrm{subject}\;\mathrm{to}\;\theta_{r}\geq 0,r=1,\ldots ,d,$$

where $z=y-\left(1/2\right)n\lambda_{0}c-\alpha 1_{n}$. Therefore, by iterating Equations (6) and (7), the intercept α and the functional components $f_{r}$ can be estimated via the penalized constrained least squares fitting framework in [45,46].

Based on the formulations above, an alternately updating strategy is proposed to find the solution of the proposed model as shown in Algorithm 1. The root mean square errors (RMSEs) from the cross-validation is used to select $\lambda_{1}$ and $\lambda_{2}$. In Algorithm 1, first, the model coefficient θ will be initialized as an all-ones vector. To determine the tuning parameter $\lambda_{0}$, a 5-fold cross-validation strategy is employed when solving the smoothing spline problem as shown in Equation (6) [43]. The selected $\lambda_{0}$ will be fixed in all later steps. The model coefficient $\beta_{r}$ will be initialized via ridge regression [47]. Next, an alternately updating strategy is used to iteratively update the coefficients based on Equations (3) and (4) to find the solution for the proposed model. The solution for Equation (3) can be obtained by iteratively update α, c and θ as shown in Equations (6) and (7). Then, by fixing α and $f_{r}$, the model coefficient $\beta_{r},r=1,\cdots ,d$ in Equation (4) can be efficiently estimated via the coordinate descent algorithm [48]. This alternately updating will be implemented until the improvement of the model performance (i.e., the RMSE) is less than the tolerance (i.e., $1\times 10^{-6}$).
**Algorithm 1** Block Updating Algorithm**Input:** data $(x_{i1},x_{i2},\ldots ,x_{id},y_{i}),i=1,\cdots ,n$; where $x_{ir}={\left(x_{ir1},\ldots ,x_{irp_{r}}\right)}^{T}$ is the *r*-th modality for product *i* with $p_{r}$ number of features**Initialization:**$\theta =1_{d}$; $\lambda_{0}$: solving the smoothing spline problem as [43], and tuning $\lambda_{0}$ according to cross-validation; $\beta_{r}$: initialized via ridge regression, r=1,⋯,d.
**Repeat** Select the tuning parameter $\lambda_{2}$ based on cross-validation **Repeat until**
α, c, and θ coverage:  Step 1: $\underset{\alpha ,c}{\mathrm{argmin}}{\left(y-\alpha 1_{n}-R_{\theta }^{*}c\right)}^{T}\left(y-\alpha 1_{n}-R_{\theta }^{*}c\right)+n\lambda_{0}c^{T}R_{\theta }^{*}c$  Step 2: $\underset{\theta }{\mathrm{argmin}}{\left(z-G\theta \right)}^{T}\left(z-G\theta \right)+n\lambda_{2}\sum_{r=1}^{d}\theta_{r},\;\;\mathrm{subject}\;\mathrm{to}\;\theta_{r}\geq 0,r=1,\ldots ,d.$ Select the tuning parameter $\lambda_{1}$ based on cross-validation **Repeat until**
$\beta_{r}$ coverage:  Step 1: $\underset{\beta_{r}}{\mathrm{argmin}}\sum_{i}{\left(y_{i}-\alpha -\sum_{r=1}^{d}f_{r}\left(x_{ir}^{T}\beta_{r}\right)\right)}^{2}+\lambda_{1}\sum_{r}{∥\beta_{r}∥}_{1}.$

## 4. Simulation

### 4.1. Simulation Setting

The objective of this simulation study is to efficiently evaluate the statistical performance (i.e., prediction accuracy and cost-effectiveness of variable selection) of the proposed model compared with other benchmark models. Specifically, this simulation study is to validate the robustness of variable selection performance with redundant predictors in each modality. The redundancy among sensor modalities will be demonstrated in the real case study. In total, eight different simulation settings are summarized in Table 1. Specifically, the sample size for each simulation case represents how many samples are generated. In each sample, the multi-modal data and the corresponding model response are generated based on a pre-defined model structure. The Decibels signal-to-noise ratio (SNR) is defined as ${\mathrm{SNR}}_{\mathrm{dB}}=10\log_{10}\left(\frac{M_{\mathrm{signal}\;}}{M_{\mathrm{noise}\;}}\right)$, where $M_{\mathrm{signal}}$ is the mean of signal power for multi-modality data, and $M_{\mathrm{noise}}$ is the power for the noise. The sparsity represents the ratio between the total significant variables and the total number of variables in the model. Finally, we chooses linear and nonlinear structures to test the robustness of the proposed methods to model a nonlinear system.

**Figure 2:**
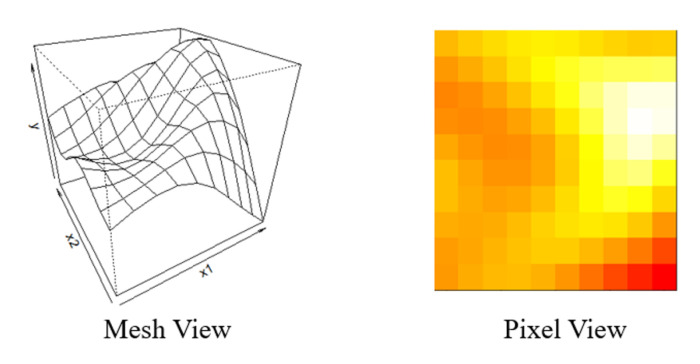
The simulated thermal distribution Signal.

To explicate the advantages of the proposed method, in each simulation, four modalities of data are generated as the raw signals. The summary of these four data modalities and the number of their corresponding features are shown in Table 2. Specifically, Modality 1 and Modality 2 are time series signals generated respectively from AR(2) model with $\phi_{1}={\left[0.9,-0.2\right]}^{T}$ and AR(3) model with $\phi_{2}={\left[-0.7,0.3,0.1\right]}^{T}$ [49]. Moreover, the i.i.d noise for both AR models is generated from N(0, 0.5). In practice, the features generated from the raw signal are widely used as predictors in modeling to reduce the data dimension and decrease the computation intensity [28]. Therefore, to effectively generate the signal features from Modality 1 and Modality 2, the discrete wavelet analysis is employed because it can effectively extract the features from both time and frequency domain [50]. Moreover, $x_{1}$ and $x_{2}$ are the features that are the Level1 and Level2 db4 detailed wavelet coefficients from Modality 1. Similarly, $x_{3}$ and $x_{4}$ are Level1 and Level2 db4 detailed wavelet coefficients extracted from Modality 2. Moreover, since there might be a 2-D image signal in the smart manufacturing system, such as a thermal distribution image, we also generate the 2-D image as Modality 3 in each sample. Specifically, the 2-D image is generated from a multivariate normal distribution, and the covariance function defined by inverse exponential squared Euclidean distance: $\Sigma \left(z,z^{\prime }\right)=\exp\left\{-{∥z-z^{\prime }∥}^{2}\right\}$ [51]. *z* is an arithmetic sequence from 0 to 2 with 10 elements. An example of the image generated in the simulation is shown in Figure 2. Moreover, $x_{5}$ and $x_{6}$ are Level1 (i.e., high-resolution image features) and Level2 (i.e., low-resolution image features) 2-D sym4 wavelet coefficients extracted from Modality 3. As disturbances, we also generate Modality 4 as the noise feature to validate the robustness of variable selection performance for the proposed model. The corresponding feature $x_{7}$ for Modality 4 is generated from a Gaussian distribution N(0,1) that has the same numerical range as the wavelet features in Modality 1–3.

After generating the features from each data modality, we need to determine the significant modalities and corresponding significant features in each sample. The significant modalities and the features will be randomly selected from Modalities 1 to 3 following a uniform distribution. Moreover, for each significant variable $x_{i,j}$ (i.e., *j*th variable from ith modality), the corresponding model coefficients $\beta_{i,j}$ is generated through a uniform distribution as Unif(−3,3). Therefore, for the simulation that has a linear model structure, the response *y* for each sample can be generated as:

(8) $$y=\sum_{i}\sum_{j}\beta_{i,j}x_{i,j}+\xi .$$

Moreover, for the simulation that has a nonlinear model structure, the response is generated as:

(9) $$y=\sum_{i}\sum_{j}\beta_{i,j}\exp\left(x_{i,j}\right)+\xi ,$$

where $\xi ∼N\left(0,\gamma^{2}\right)$, and the magnitude of $\gamma^{2}$ is determined by the signal-to-noise ratio from the simulation setting.

For each simulation setting shown in Table 1, 100 replicates are simulated. The proposed MOSS is compared with three benchmark models to evaluate its prediction performance and also the variable selection accuracy: (1) the Lasso regression which can only implement the variable selection efforts without the concept of data modality [24]; (2) the group Lasso which can implement the variable selection in modality level but cannot select the variable within each modality [37]; and (3) the hierarchical Lasso which can implement the variable selection in both among modalities and within each modality [26]. These three benchmarks can help to comprehensively validate the performance of the MOSS for both variable selection and prediction accuracy. To evaluate the prediction accuracy, in each replication of the simulation, 80% samples are used as the training data set, and the remaining 20% of samples are used as the testing data set. To fairly compare the variable selection accuracy, the significant variables for each simulation case are the same among each replication. Moreover, the number of modalities selected from the MOSS is fixed as the maximum number of modalities selected among benchmarks in each replication. Based on this scenario, we can validate whether the proposed MOSS can effectively guide the multi-modal sensing system design with a limited budget (i.e., limited sensor modalities) by selecting the most relevant sensor modalities compared with benchmarks.

### 4.2. Results and Discussion

The average of the normalized root-mean-squared error (RMSE) and the corresponding standard error for eight simulation cases are shown in Table 3. The values shown in bold are the smallest prediction errors and the corresponding standard error obtained from different models in each simulation case. From the results, the proposed MOSS yields the best prediction accuracy in most of the cases with both linear and nonlinear model structures. It is because the proposed MOSS can deal with the nonlinear model components, and can effectively implement the variable selection for both among the modalities and within each modality compared with the benchmarks via the function norm and *l*-1 norm simultaneously. For the Lasso regression, it can be observed that the standard error is relatively large than other methods. It is because without considering the variable relationships among modalities, the variable selection result might not be stable among replications. Moreover, since the group Lasso cannot effectively implement the variable selection within each modality, more insignificant variables are included in the model and the prediction accuracy is relatively low. For the hierarchical Lasso, it has a comparable result with the proposed MOSS method, but for the nonlinear model components, the proposed MOSS has a better prediction accuracy since the functional norm can work with both linear and nonlinear model components.

On the other hand, to evaluate the variable selection accuracy of each method, the Recall is employed as the performance measurement:

$$\mathrm{Recall}=\frac{\;\mathrm{Number}\;\mathrm{of}\;\mathrm{Significant}\;\mathrm{Variables}\;\mathrm{Selected}\;}{\;\mathrm{Total}\;\mathrm{Number}\;\mathrm{of}\;\mathrm{Selected}\;\mathrm{Variables}\;}.$$

It is because the Recall can reasonably reflect the cost-effectiveness of variable selection results. The results are shown in Table 4. The proposed MOSS yields the best cost-effective performance in all simulation settings. It shows the merits of the proposed MOSS that can efficiently select the significant modalities and variables simultaneously. Moreover, the group Lasso has good precision for most simulation cases. The Lasso regression almost has the worst variable selection performance on all simulation settings since it cannot address the modality structure among variables, and can only consider the variables that are independent in variable selection. Moreover, it is not surprising since the group Lasso does not implement the variable selection within each modality, therefore the number of selected variables for group Lasso is much higher than other methods. The recall for the group Lasso also proves this idea. The hierarchical Lasso usually has a comparable variable selection precision with the MOSS since it can also implement the variable selection for both modalities and within each modality. But limited by its linear model component assumption, the proposed MOSS can be more flexible compared with the hierarchical Lasso.

## 5. A Real Case Study

### 5.1. Experiment Setup

**Figure 3:**
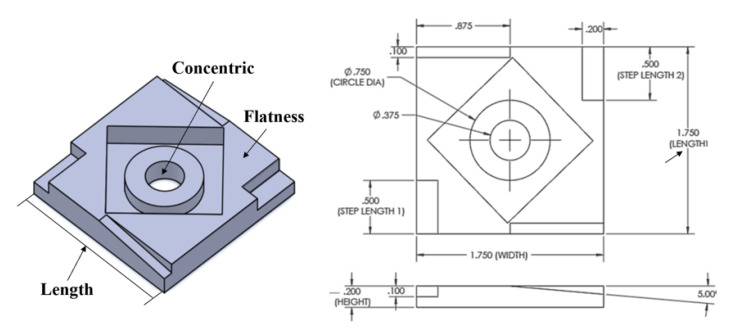
Standard drawing of NAS 979 part (unit: inch) [10].

In order to evaluate the performance of the proposed model, we apply the proposed method to the data sets collected from a real FDM system showed in Figure 1 [18]. In the data sets, the in situ process variables collected from different sensor modalities, and the geometric quality variables measured from the coordinate-measuring machine for each product were organized to model the corresponding quality-process relationship. Specifically, in the data sets, 48 products were fabricated based on a full factorial design with three replications. The modified national aerospace standard 979 test part design (as shown in Figure 3) is selected as the product design [10]. The selected process setting variables in the experiment are shown in Table 5. In total, there are four setting variables in two levels: extruder speed, extruder temperature, temperature disturbance, and platform vibration disturbance. The full design of the experiment table is attached in Appendix A Figure A1. The extruder speed and the extruder temperature are both the significant setting variables that can directly influence the product quality [52]. To introduce extra disturbance to the system, two types of process noise are involved in the experiment. The disturbances are introduced by a fan near the extruder, which can significantly change the thermodynamic in the near area, and a vibrator with a fixed frequency on the printing bed. This vibrator can provide a periodic impetus to the accelerometer that was equipped on the printing bed. Therefore, it can introduce a non-stationary signal component to the signal collected from the accelerometer. These disturbances are employed during the fabrication process, and expect to validate whether the proposed method can identify the disturbance in variable selection results.

The multi-modal sensing system for the FDM process in the experiment is shown in Figure 1. It is equipped with two tri-axis accelerometers, two thermocouples, and one infrared (IR) sensor. The infrared sensor and the thermocouple on the extruder can generate correlated raw signals and introduce the redundant data to this system. All signals are measured at a sampling frequency of 1 Hz via a data acquisition system built by Ni-cRIO-9073. Such a sensor selection and frequency combination has shown to be effective to reflect the real-time FDM process condition [10,12]. For the vibration sensor, it contains the vibration signals from the two-axis, and each axis is considered as one separate data modality. It is because the signal from each axis can reflect different types of process variation for a FDM process, and can further help to accurately identify the significant modality in the process. The wavelet analysis is used to compactly represent the in situ signals collected from these sensors in this case study. Specifically, the Level4 detail wavelet coefficients generated based on the db4 basis are employed as signal features in this case study. Finally, there are 47 features extracted from each data modality, and there are nine data modalities in total. After the product fabrication, the coordinate measuring machine is used to measure the corresponding geometric quality variables (i.e., length, flatness, and concentric).

### 5.2. Results and Discussion

To evaluate the prediction performance of the proposed model, a 5-fold CV training-testing strategy is employed. Similar to the simulation study, the Lasso, group Lasso, and hierarchical Lasso are used as the benchmark methods. The average of normalized RMSEs for testing from 5-fold CV is shown in Table 6. It can be observed that the proposed MOSS yields the best prediction accuracy for all three quality measurements. It is because the proposed method can properly identify the significant data modalities based on the smooth spline functional norm and also the important features within each modality. The Lasso regression has the worst prediction accuracy since it does not consider the modality structure among each variable. This issue might lead to an inaccurate variable selection result. Similarly, the group Lasso has comparable results with the Lasso regression since it can only consider the variable selection among modalities. Moreover, the hierarchical Lasso has a better result compared with Lasso and group Lasso since it can implement the variable selection on two-levels simultaneously. However, due to it might usually restrict on a local optimal when estimating the model coefficients, the proposed method could be more effective to identify the significant modalities.

**Figure 4:**
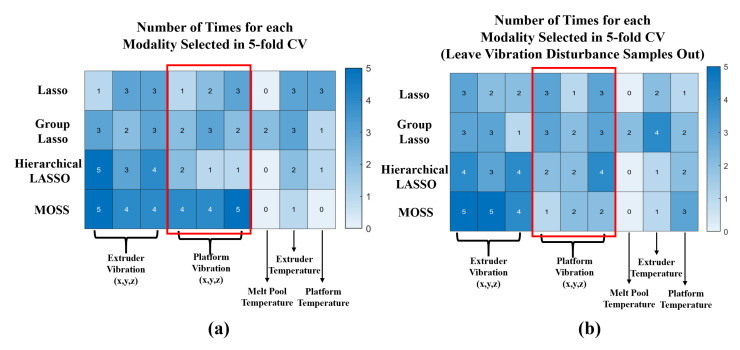
(**a**) Number of Times for each Modality Selected in 5-fold CV; (**b**) Number of Times for each Modality Selected in 5-fold CV after Leaving Vibration Disturbance Samples Out.

On the other hand, to evaluate the modality selection results, the number of times that each modality is selected in the 5-fold CV for product flatness is shown in Figure 4. Specifically, the modality selection results for two scenarios are studied: (1) the modality selection results with all samples collected from the experiments; and (2) the modality selection results for the samples that do not have the vibration disturbance on the printing bed. The motivation of this sensitivity analysis is to evaluate whether the proposed method and the benchmarks can accurately identify the significant data modalities in model estimation. In Figure 4, it can observe that when the printing platform has the vibration disturbance, the proposed MOSS method can effectively identify the influence of extruder and platform vibration in the model estimation, which are the most relevant modalities for product flatness [12]. Once the vibration disturbance is removed, the number of selection times for platform vibration is significantly reduced. It is because the contribution of platform vibration is decreasing without the vibration disturbance during the fabrication process. On the other hand, other benchmarks cannot always select these important modalities in model estimation. Moreover, after removing the samples that have the vibration disturbance, the proposed MOSS method can also effectively identify the most relevant modalities (i.e., extruder vibration) in this scenario and have a better selection accuracy compared with other benchmarks in a 5-fold CV. Therefore, it can be concluded that the proposed MOSS can effectively select the sensing modalities in a quality model. This result can further guide the multi-modal sensing system design and support the root cause analysis to improve the product quality and the process reliability of the FDM.

**Figure 5:**
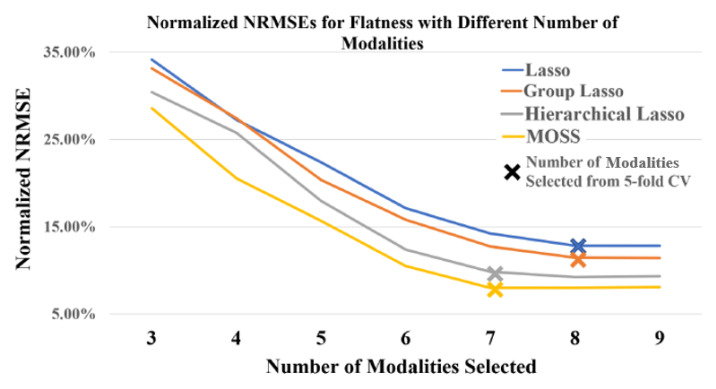
Normalized NRMSEs for Flatness with Different Number of Modalities.

**Figure 6:**
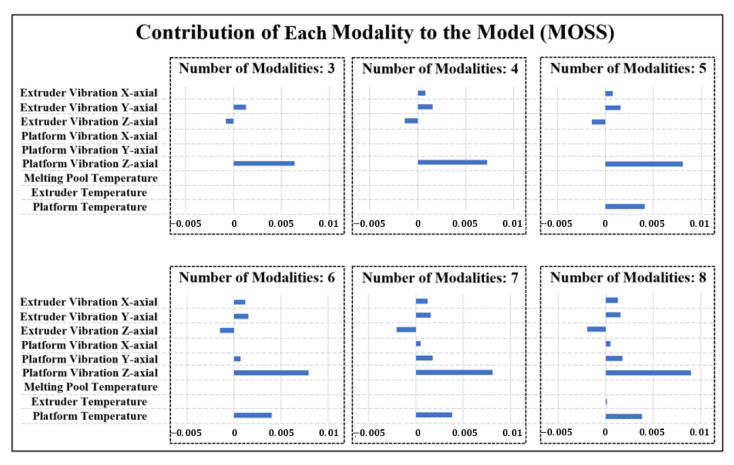
Contribution of each Modality for Flatness Prediction with Different Numbers of Modalities in MOSS.

**Figure 7:**
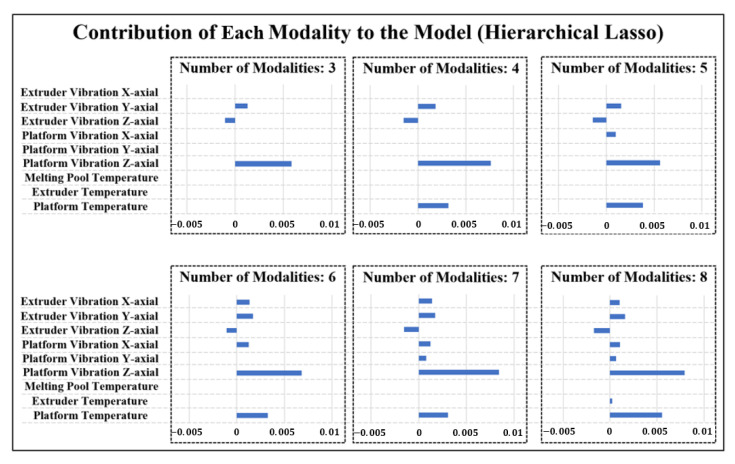
Contribution of each Modality for Flatness Prediction with Different Numbers of Modalities in Hierarchical Lasso.

Moreover, to identify whether the proposed MOSS can effectively identify the best subset of modalities when modeling the quality-process relationship, the prediction results for product flatness with a different number of selected modalities are shown in Figure 5, Figure 6 and Figure 7. The number of modalities selected represents the maximum number of modalities that the method can select in model estimation. To guarantee the modeling performance, the number of selected modalities is started from three. It can be observed that in Figure 5 the proposed MOSS method yield the best prediction accuracy in all scenarios compared with benchmarks. It is because that the proposed MOSS method can accurately select the significance of the sensing modality. To validate this point of view, we also summarized the selected modalities in detail. Due to the limited space, we mainly showed the selected modalities for MOSS and Hierarchical Lasso in Figure 6 and Figure 7 for the number of modalities from three to eight. Since the hierarchical Lasso has the closest prediction accuracy with the MOSS. Based on the modality selection result, it can be observed that the proposed MOSS can accurately select the modalities in a proper order compared with the benchmark. For example, when the number of selected modalities increased to four, the MOSS selected x-axial extruder vibration as the additional modality, and the hierarchical Lasso selected platform temperature as the additional modality. For the flatness of the product, as discussed above, the variation of platform temperature is not significant compared with the vibration on the extruder. This modality selection result also explains why the prediction accuracy for Moss is much better than hierarchical Lasso when the number of selected modalities is four. On the other hand, it can also be found that even though the selected modalities are the same for both MOSS and hierarchical Lasso, the prediction accuracy of Moss is still slightly better than the hierarchical Lasso. One possible explanation is the MOSS can better leverage the selection efforts between the modalities and the variables within each modality based on the smooth spline non-parametric estimation. Moreover, the hierarchical Lasso usually yields a local optimal due to the modeling estimation restriction [27]. The MOSS also has the flexibility to control the number of modalities selected in the model estimation, and further guide a cost-effective multi-modal sensing system design. Therefore, when there are limited resources and have to select the best subset of modalities, the MOSS can still select the most relevant modalities, and while estimating an accurate quality-process model.

## 6. Conclusions

Smart manufacturing integrates the multi-modal sensing system and the computation capability to effectively support real-time data analytics. However, how to design a multi-modal sensing system with a cost-effective consideration for the manufacturing process is a challenging question. Because it is difficult to accurately identify the relevance and contribution of each sensor modality toward the specific quality response. Therefore, in this research, we proposed a new model called MOSS, which can effectively rank the significant sensor modalities and simultaneously identify the important features within each modality in model estimation. It can guide the sensing system design in smart manufacturing, and also provides a way to identify the contribution of each modality to potentially guide the diagnosis for the quality variation [10]. The MOSS can be easily extended to other applications and domains, such as other manufacturing processes or healthcare applications which usually need to model the data with a multi-modal format [53,54].

This research also leads to several future research directions. First, we can generalize the MOSS so that multiple quality responses can be jointly modeled. One possible extension of the MOSS is to multiple response regression under the non-parametric estimation framework [55]. Next, the spatial process variables and quality responses, such as the thermal video and 3d profile of the product, can be incorporated into the MOSS to reasonably quantify the spatio-temporal relationship contained in both process variables and quality variables [56,57]. Finally, the monitoring and control strategy can also be integrated with the MOSS in a real-time manner to effectively detect the anomaly event during the fabrication process, and further improve process reliability and reduce process variation [58].

## Figures and Tables

**Table 1:** Simulation Settings.

Case No.	Sample Size	Signal-to-Noise Ratio (db)	Sparsity (Total Significant Variables in All Modalities)	Model Structure
1	100	1	0.1 (6)	Linear
2	100	0.6	0.25 (16)	Nonlinear
3	100	1	0.1 (6)	Nonlinear
4	100	0.6	0.25 (16)	Linear
5	300	1	0.1 (6)	Nonlinear
6	300	0.6	0.25 (16)	Linear
7	300	1	0.1 (6)	Linear
8	300	0.6	0.25 (16)	Nonlinear

**Table 2:** Data Summary in Simulation (number of features is shown in parenthesis).

Data	Modality 1		Modality 2		Modality 3		Modality 4
Features	High-resolution time-series features (8)	Low-resolution time-series features (4)	High-resolution time-series features (8)	Low-resolution time-series features (4)	High-resolution image features (25)	Low-resolution image features (4)	Noise generated from Normal distribution (11)

**Table 3:** Normalized Root-Mean-Square Error (RMSE) (Standard Error) of Each Simulation Case.

	Lasso Regression	Group Lasso	Hierarchical Lasso	MOSS (Proposed)
Case 1	8.72% (0.04)	8.35% (0.02)	8.37% (0.02)	**7.58%** **(0.02)**
Case 2	9.42% (0.07)	9.10% (0.01)	8.82% (0.02)	**7.71%** **(0.01)**
Case 3	15.75% (0.05)	14.97% (0.03)	13.24% (0.05)	**11.92%** **(0.02)**
Case 4	9.94% (0.05)	9.51% (0.02)	8.42% (0.02)	**7.93%** **(0.02)**
Case 5	13.41% (0.06)	12.75% (0.04)	12.69% (0.05)	**10.46%** **(0.02)**
Case 6	7.81% (0.07)	7.04% (0.01)	7.15% (0.01)	**6.67%** **(0.01)**
Case 7	8.65% (0.06)	8.17% (0.01)	7.81% (0.04)	**7.23%** **(0.02)**
Case 8	12.89% (0.08)	10.61% (0.01)	10.17% (0.02)	**8.82%** **(0.02)**

**Table 4:** Average Variable Selection Recall of Each Simulation Case.

	Lasso Regression	Group Lasso	Hierarchical Lasso	MOSS (Proposed)
Case 1	51.2%	56.2%	61.2%	**64.8%**
Case 2	54.3%	57.1%	62.3%	**68.7%**
Case 3	55.1%	60.8%	63.3%	**61.9%**
Case 4	48.2%	52.4%	62.9%	**68.7%**
Case 5	60.2%	54.1%	70.4%	**75.4%**
Case 6	63.4%	58.7%	68.6%	**73.2%**
Case 7	66.1%	59.2%	67.3%	**71.5%**
Case 8	63.2%	53.8%	70.6%	**73.6%**

**Table 5:** Setting Variables in the Experiment [18].

	Extruder Travel Speed	Extruder Temperature	Temperature Disturbance	Vibration Disturbance
Level 1	40 mm/s	$225^{\circ }$	On	On
Level 2	70 mm/s	$245^{\circ }$	Off	Off

**Table 6:** Average of Normalized RMSEs.

Quality Measurements (from CMM)	Lasso	Group Lasso	Hierarchical Lasso	MOSS (Proposed)
Length	20.15%	17.65%	16.14%	**14.57%**
Flatness	12.43%	11.44%	9.79%	**7.91%**
Concentric	11.06%	9.83%	9.02%	**7.86%**

## Data Availability

The data presented in this study are available on request from the corresponding author.

## Appendix A

**Table A1:** Design of Experiment Table for Case Study [12].

Run Number	Number	Extruder Speed Level	Extruder Temperature Level	Temperature Disturbance Level	Vibration Disturbance Level
44	1	0	0	0	0
43	2	0	0	0	1
7	3	0	0	1	0
48	4	0	0	1	1
20	5	0	1	0	0
21	6	0	1	0	1
6	7	0	1	1	0
29	8	0	1	1	1
12	9	1	0	0	0
26	10	1	0	0	1
30	11	1	0	1	0
24	12	1	0	1	1
14	13	1	1	0	0
22	14	1	1	0	1
3	15	1	1	1	0
38	16	1	1	1	1
10	17	0	0	0	0
28	18	0	0	0	1
33	19	0	0	1	0
41	20	0	0	1	1
32	21	0	1	0	0
8	22	0	1	0	1
15	23	0	1	1	0
45	24	0	1	1	1
19	25	1	0	0	0
36	26	1	0	0	1
42	27	1	0	1	0
35	28	1	0	1	1
11	29	1	1	0	0
31	30	1	1	0	1
5	31	1	1	1	0
4	32	1	1	1	1
16	33	0	0	0	0
1	34	0	0	0	1
13	35	0	0	1	0
40	36	0	0	1	1
2	37	0	1	0	0
39	38	0	1	0	1
46	39	0	1	1	0
25	40	0	1	1	1
34	41	1	0	0	0
23	42	1	0	0	1
17	43	1	0	1	0
37	44	1	0	1	1
27	45	1	1	0	0
47	46	1	1	0	1
18	47	1	1	1	0
9	48	1	1	1	1
